# Mitochondrial Gene Phylogenetic Incongruencies Are Linked to Chromosomal Position and Function

**DOI:** 10.1093/gbe/evaf209

**Published:** 2025-11-10

**Authors:** Rob DeSalle, Michael Tessler

**Affiliations:** Institute for Comparative Genomics, American Museum of Natural History, New York, NY 10024, USA; Division of Invertebrate Zoology, American Museum of Natural History, New York, NY 10024, USA; Institute for Comparative Genomics, American Museum of Natural History, New York, NY 10024, USA; Division of Invertebrate Zoology, American Museum of Natural History, New York, NY 10024, USA; Department of Biology, Medgar Evers College, Brooklyn, NY 11225, USA

**Keywords:** mitochondrial genes, incongruence, light strand, mutational bias

## Abstract

Mitochondrial DNA has been one of the key workhorses of evolutionary studies. Hence, understanding the dynamics of DNA sequence change in this tiny genome (15 to 20 kb) is of utmost importance. However, we are unaware of large studies examining how the functionality and chromosomal positioning of mitochondrial genes may impact their phylogenetic patterning. To examine this, we assembled a large database of animal mitochondrial genomes (>10,000 total individuals over 89 taxonomic groups) and compared their phylogenetics, functionality, and location on the mitochondrial genome (heavy and light strand in vertebrates or J and N strand in other animals and distance from the origin of replication). We found that many genes show unique evolutionary patterns, often directly tied to chromosomal location or gene function (eg NADH dehydrogenases or ribosomal RNA genes). We also found rampant phylogenetic incongruence among the linked genes of the mitochondria in most of the taxonomic groups we examined. These results suggest that mitochondrial genomes have accrued complex evolutionary patterns. The accumulated incongruence can influence phylogenetic inference in evolutionary studies, making mitochondrial gene choice for phylogenetics critical. The phenomena we show here should also be examined in other organelle and even nuclear gene studies.

Significance Mitochondrial DNA has been used in evolutionary studies now for over 50 years. Specifically, it has been used as a source of phylogenetic information in systematics. This study leverages over 10,000 whole animal mitochondrial genomes from a diverse set of taxonomic groups to compare the phylogenetic signal in the 13 protein coding and 2 ribosomal RNA (rRNA) genes of a typical animal mtDNA genome. Our study reveals that there is a large degree of phylogenetic incongruence among these genes when compared for phylogenetic signal for most of the taxonomic groups we examined. We also show that phylogenetic incongruence patterns show unique evolutionary patterns often correlated to location on the circular mtDNA molecule, strand location, or function.

## Introduction

The dynamics of mitochondrial genome change have been at the heart of evolutionary analysis for four decades (Brown et al. 1979; Avise 1986; Wallace 1994; Clayton 2000; Cameron 2014; DeSalle and Hadrys 2017; DeSalle et al. 2017). Since early use as a phylogenetic tool (Crozier 1990; Hiesel et al. 1994; Simon et al. 1994; Boore and Brown 1998; De Mandal et al. 2014; DeSalle and Hadrys 2017; DeSalle et al. 2017), mitochondria have been utilized for understanding natural selection (Blier et al. 2001; Rand 2001; Ballard and Whitlock 2004; Ballard and Rand 2005; Garvin et al. 2015; Towarnicki and Ballard 2020), examining mitochondrially inherited human diseases (Wallace 1994; Taylor and Turnbull 2005; Tuppen et al. 2010; Wallace 2018; Javadov et al. 2020), and as a marker for DNA barcoding (Hebert et al. 2003; Hebert and Gregory 2005; Antil et al. 2023). In short, this molecule has been a major player in how we perceive evolutionary patterns and processes. Because of these many studies of mitochondrial DNA, mitogenome sequencing has proliferated (Curole and Kocher 1999; Bernt et al. 2013a, b; Cameron 2014; Crampton-Platt et al. 2016; Zardoya 2020). These databased sequences offer an unprecedented opportunity to examine how this haploid, clonally inherited, mostly maternally inherited genome has evolved in eukaryotic organisms.

Variation in mitochondrial genomes has been examined in detail. For instance, gene order rearrangement has been used to understand deep phylogenetic resolution (Boore et al. 1995; Dowton et al. 2002; Lavrov and Lang 2005; Bernt et al. 2013a, b; Lavrov and Pett 2016), while the evolutionary generation of point mutations in specific genes—most notably, cytochrome oxidase 1 (COX1) for DNA barcoding of animals—has been the target of many studies. Hypotheses concerning the nature of variation at the sequence level have been examined, as well as studies of how the molecule replicates and self corrects (Clayton 2003; Falkenberg et al. 2007; Sahyoun et al. 2014; Yasukawa and Kang 2018; Xia 2019).

The mitochondrial molecule in most animals is circular and double stranded, with extremely reduced gene content (13 protein coding genes, 2 rRNAs, and usually 22 tRNA genes; Wolstenholme 1992; Taanman 1999). In the vertebrates, the two strands of the molecule have been designated “light” and “heavy” based on their density. The terms majority or J (juxtaposed) strand and minority or N (non-juxtaposed) strand are used in insects and other invertebrates (broadly defined). In this latter terminology, the majority strand (J) carries the majority of genes in the genome while the minority (N) carries the minority of genes. This terminology results in difficulty in homologizing the strands between vertebrates and say insects. Hence, for the bulk of this study to standardize our discussion with respect to strandedness, we will refer to the vertebrate heavy strand as the majority strand and the vertebrate light strand as the minority strand.

**Fig. 1.**
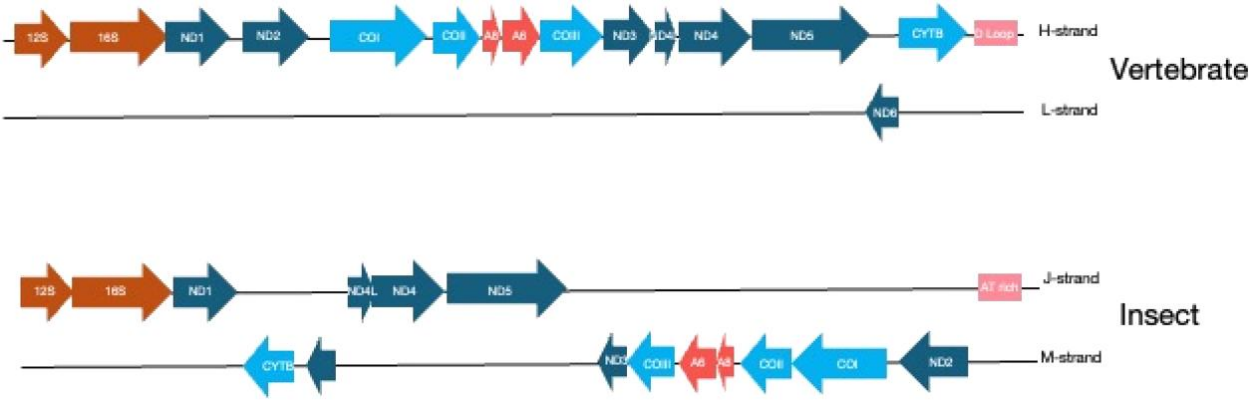
Generalized gene order maps of mitochondrial DNA of vertebrates (top) and insects (bottom). The arrows indicate the direction of transcription and strand location (rightward arrows indicate heavy strand and leftward arrows indicate light strand). Functional categories are also indicated in the diagram: brown = rDNA (12S and 16S); light blue = cytochrome genes (COX1, COX2, COX3, and CYTb); orange = ATPases (ATP6 [A6] and ATP8 [A8]); dark blue = NADH dehydrogenases (ND1, ND2, ND3, ND4, ND4L [4L], ND5, and ND6.

Different lineages of animals have distinct arrangements of these genes on the two strands, which are replicated in different directions and through different molecular processes (Clayton 2003). In general, one strand is replicated continuously and the other is replicated using strand displacement (Zheng and Shen 2011; Yasukawa and Kang 2018). For most vertebrates, only one protein coding gene (ND6) is on the light strand and the rest are on the heavy strand. While there is ample variation in the arrangement of mitochondrial genes in other animals, we show the general derived insect arrangement as a standard (Fig. 1). Most insect mtDNA has a somewhat even distribution of genes on both the majority and minority strand, but a rather diverse array of gene order on these strands exists for other invertebrates. In addition, this highly reduced mitogenome has three major functional categories of protein coding genes—NADH dehydrogenases, ATP synthases (AT), and cytochrome oxidases (Hebert et al. 2003).

A recent study of the phylogenetic signal in the genes of the mitogenomes of flies in the family Drosophilidae demonstrated that a number of these genes had incongruent phylogenetic patterns across the drosophilid phylogeny (DeSalle et al. 2022). While some incongruence is expected among linked genes as a result of different mutation and selection regimes in the various genes in the drosophilid mitogenome, the disagreement was widespread. This prompted us, here, to examine a broader swath of animal taxa to see how widespread the incongruence might be and what mechanisms might be at play in the evolution of animal mitochondrial DNA.

Because of the distinct variation in the distribution of the 13 protein coding mitochondrial genes in different animals, we also have a unique opportunity to examine if structural changes (like gene order or rearrangement of genes from one strand to the other) are involved in generating the incongruent phylogenetic signals that were observed in the Drosophilidae (DeSalle et al. 2022). As shown in this paper, all groups of animals with extensive mitogenome sequencing also show these patterns of incongruence in phylogenetic analysis in one way or another.

This paper attempts to (i) quantitate the degree of incongruence of mitogenome protein coding genes and rRNA genes across animal groups and (ii) examine any correlation of the biology of animal genomes with the phylogenetic patterns we see. As for the latter goal, we are mostly interested in gene arrangements, strand location of the genes, and how the different replication mechanisms of the majority and minority strands of the genome might influence the phylogenetic signal of mitochondrial genes in different animal groups.

## Results

### Taxonomic Spread

The datasets we amassed are distributed over much of animal life. To present the results we use some higher order group comparisons that are not implied to be phylogenetically meaningful like “invertebrate versus vertebrate.” By vertebrate, we mean all taxa with vertebrae, which are also all deuterostomes. There are some deuterostomes without skeletal vertebrae—Echinodermata and *Branchiostoma*, which we examine with respect to their vertebrate/invertebrate status. In addition, due to the presence of invertebrate lineages outside of Bilateria, monophyly of invertebrates is also disturbed and hence lack of invertebrate monophyly needs to be considered. The 89 nexus matrices are available on Data Dryad (DOI: 10.5061/dryad.m0cfxpp9m).

### Comparing Tests of Incongruence

For each gene partition, we obtained a matrix of the significance values from the bp-RELL, p-KH, p-SH, p-wKH, p-wSH, c-ELW, p-AU (all defined in by Nguyen et al. 2015), and the p-incongruence length difference (ILD) analyses (p stands for p value and w stands for weighted estimate) for each pairwise comparison. It was clear from this preliminary analysis (not shown) that the different measures have differing degrees of sensitivity in inferring congruence. For instance, the unweighted SH test is extremely sensitive and infers congruence at a much higher rate than any of the other tests. The AU test appears to be less sensitive, as it infers congruence at much lower rates than the SH test. This is consistent with studies that have shown that the weighted SH and KH tests, as well as the AU test, are more conservative and hence could be more appropriate for inferring congruence. This preliminary analysis suggests that a viable approach to comparing congruence with these datasets would be to focus on the weighted likelihood tests and the AU test. We include the SH test for comparison.

### Dataset Size Considerations

**Fig. 2.**
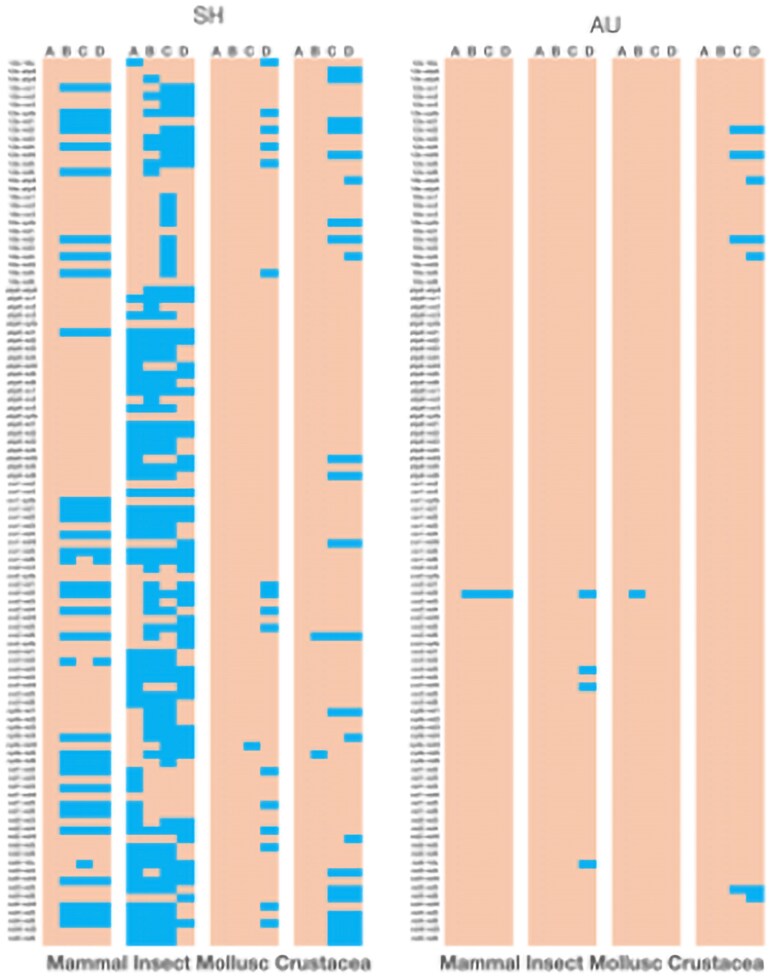
Comparison of the effect of number of taxa in a dataset on incongruence for SH and AU tests. Color scheme: blue = congruent; orange = incongruent. SH and AU refer to the likelihood test for congruence. The animal group examined is listed at the bottom. The percent of the dataset remaining is shown in columns A (100%), B (50%), C (25%), and D (10%). The pairwise gene comparison is shown to the left of each panel.

One way to assess the influence of dataset size is to take the larger datasets in our database and randomly pare them down to see if congruence measures are impacted by making smaller and smaller taxon sets. Figure 2 shows the results of reducing dataset size on congruence for both the SH and AU tests. It is clear from this figure that congruence increases as the dataset size is reduced. Except for one case (mammals), the increase in congruence is not drastic for SH or AU tests in most cases. Dataset sizes under 50 taxa sometimes show high congruence amongst gene partitions.

### General Levels of Pairwise Gene Congruence among Taxonomic Groups

**Fig. 3.**
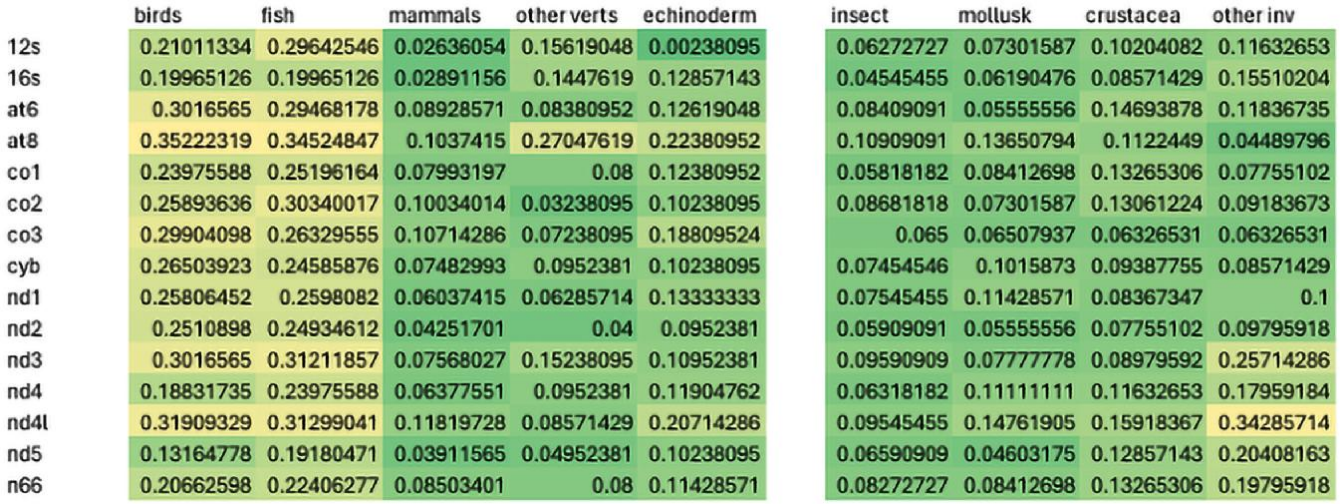
Heatmaps showing the distribution of congruence frequency by gene in the 89 datasets. Each taxonomic group was scaled separately; green indicates lower proportion of congruence while yellow indicates higher proportion of congruence. Congruence in the figure ranges from a low of about 0.04 (4%) to a high of 0.35 (35%).

Figure 3 shows a summary of the results of congruence tests for the major taxonomic groups in this study for the seven likelihood tests. For deuterostomes (both invertebrate and vertebrate), fish and birds show the highest degree of gene-by-gene congruence (25% of the total tests were congruent) followed by echinoderms (on average about 15% congruent), other vertebrates (10%), and finally with mammals (about 7% congruent) showing the least for the deuterostomes. For protostomes and the non-bilaterian lineages (Porifera, Cnidaria, Placozoa, and Ctenophora), the levels of congruence in pairwise comparisons are generally smaller than for vertebrate deuterostomes (all in general about 5%). A close examination of Fig. 3 suggests that there is also a great deal of incongruence in gene-by-gene comparisons for mitochondrial genes with no taxa reaching more than 35% of the comparisons being congruent.

**Fig. 4.**
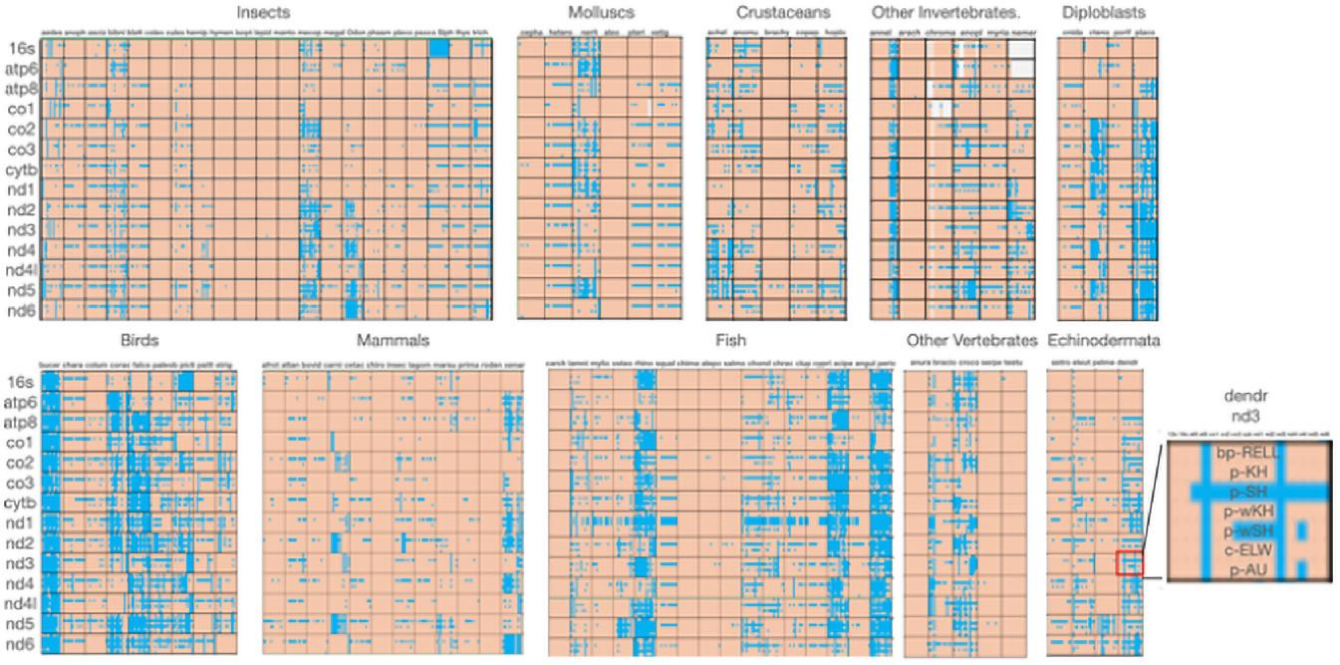
General level of pairwise congruence using seven tests of congruence significance. The major taxonomic groups are labeled at the top of each set of sub blocks. Color scheme: blue = significantly congruent; orange = congruent. The inset shows the organization of data in each of the cells in the figure. Genes compared to the genes on the far right of the figure and the seven ML tests for congruence.

Figure 4 shows a summary of the distribution of congruence measures across genes in pairwise comparisons. In general, two genes stand out as having higher than usual congruence—ATP8 and ND4L. Without surprise, these two genes are the shortest genes in the mitochondrial genome and the higher relative congruence may be due to their short lengths. At the same time, it should be pointed out that the congruent genes percentage for all comparisons are extremely low—often times being under 10%.

### Levels of Incongruence between Individual Genes and Rest of the Mitogenome

**Fig. 5.**
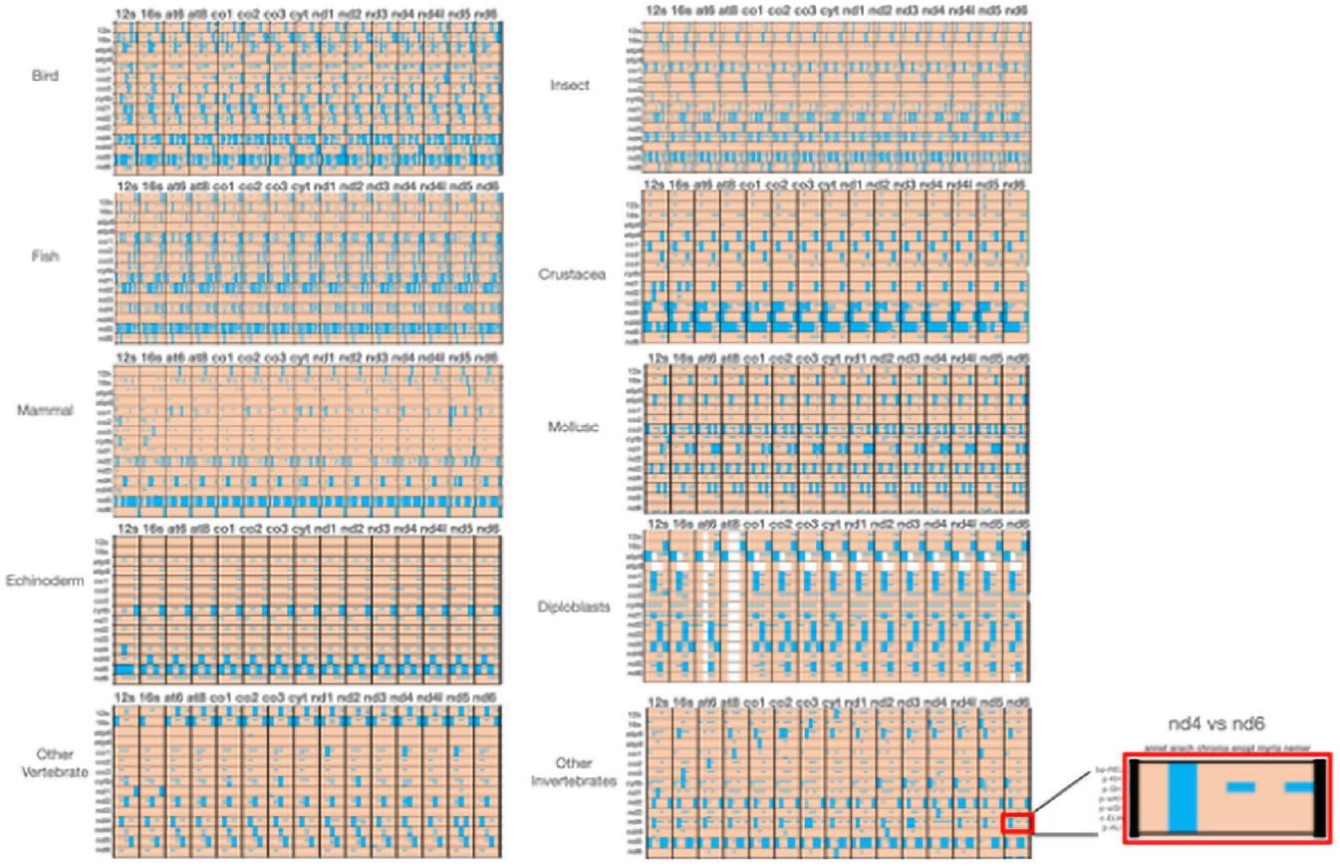
Levels of congruence within taxonomic groups upon removal of the partition listed on the *y* axis. Color scheme: blue = congruent; orange = incongruent. Each taxonomic block is comprised of a gene listed along the top, which is compared for congruence to a matrix of combined genes missing the gene listed for the row. So for instance the 12S × 12S block compares the congruence of 12S rRNA with a matrix including all genes except 12S rRNA. Similarly, the 12S rRNA × 16S rRNA block compares the congruence of the 12S rRNA gene with a matrix including all genes except 16S rRNA, and so on. The inset on the right shows where congruence information is in each cell of the figure.

We next examined the impact of removing a single data partition on the overall congruence of comparisons with single gene partitions (anti–gene-by-gene comparisons). This approach essentially compares the congruence of the whole mitogenome matrix minus a specific gene to the individual protein coding and rRNA genes. The idea here is that if removal of a gene partition from the overall dataset results in congruence with other partitions, then the removed partition contributes significantly to the incongruence in the dataset. Figure 5 shows the results of this approach.

The general result of this latter analysis is that certain genes can be highlighted as contributing incongruence relative to the rest of the genome. Any stretches of blue labeling in Fig. 5 indicate congruence gained by removal of the gene listed in the left of the blocks. It is safe to say that removal of NADH dehydrogenase (ND) genes most often produces higher degrees of congruence. Specifically, removal of ND5 in these tests results in much higher overall congruence, suggesting a major split of this gene in comparison to the rest of the genome regardless of taxonomic group. Removal of all other genes results in much smaller proportion of congruence upon removal of those genes and again ND4L and ATP8 appear to have the strongest negative effect on congruence upon removal from the dataset. This result is more than likely due to the small size of these two genes compared to the rest in the mtDNA genome.

### Incongruence between Functional Groups of Genes

**Fig. 6.**
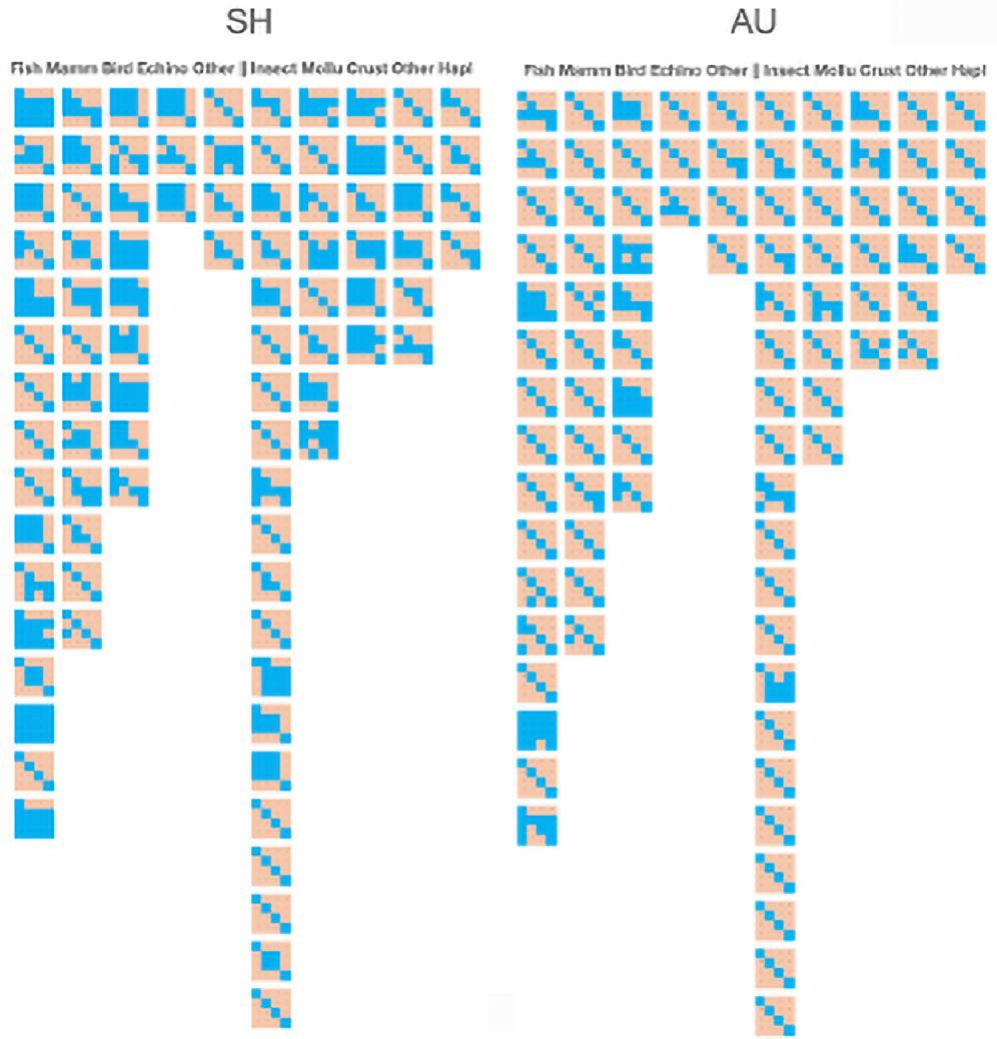
Comparison of congruence of functional categories. Results from the SH test are on the left and for the AU test on the right. Yellow indicates significant congruence and blue indicates lack of significant congruence.

We also partitioned our analysis to consider the four major kinds of functional groups of genes in the mitogenome: cytochrome oxidases (CO), ND, AT, and rRNAs. The results of this analysis are shown in Fig. 6 and demonstrate that there is a high degree of incongruence between genes in different functional categories. The only exception to this statement are birds where the functional categories are fairly congruent for both the SH and AU tests. The rRNA category is highly incongruent with the other three categories for nearly every taxonomic group and across the two tests.

### Distance from the OR and Unscaled Incongruence Measures

We compiled the distance of the 15 genes used in this study from the origin of replication (OR) for taxonomic groups where the OR is known and used these distances to test for correlation with unscaled congruence measures for the taxa in this study. These estimates are very group specific as the distribution of genes on the heavy and light (for vertebrates) or majority and minority strands (for non-vertebrates) varies among taxonomic groups and there has been considerable gene rearrangement in the genomes of many of the taxonomic groups represented here.

To get a measure of congruence for each gene, we calculated the number of significant likelihood congruence tests for each of the 89 taxa we included in this study for each gene in pairwise comparisons. This metric is simple and easy to compute and gives values that quantify the number of comparisons a specific gene partition is congruent with respect to all others. Table 1 shows the results of the regression analyses. The table demonstrates a lack of significant linear relationship of distance from the OR on the majority strand in almost all taxa (R^2^ is less than 0.2 in all cases except for Nemertea, Lepidoptera, and Mammals). Conversely, the analyses detect significant correlation of distance from the OR on the minority strand with the degree of congruence for some protostome invertebrate taxa. The members of the protostome class Insecta show consistent correlation of distance from the OR with congruence (most correlation coefficients are >0.5). Two members of protostome invertebrates—crustaceans (two groups—Achelata and Anomura), and one protostome molluscan (one group—Heterobranchia) taxon show significant correlation too.

**Table 1:** Results of regressing distance (in bp) from OR on unscaled congruence measure

H taxon	Taxon	HR	Hm	LR	Lm
Vertebrate^a^	Mammals	0.33	N	NA	NA
	Aves	0.04	N	NA	NA
	Fish	0	N	NA	NA
	All vertebrates	0.01	N	NA	NA
Other Deut	Anura	0.16	P	NA	NA
	Branchiostoma	0.23	P	NA	NA
	Crocodylia	0.02	N	NA	NA
	Serpentes	0	0	NA	NA
	Testudinata	0.13	N	NA	NA
	Echinoderm	0.12	N	NA	NA
	All Deutero	0.08	N	NA	NA
Diploblast	Porifera	0.05	N	NA	NA
	Placozoa	0.05	N	NA	NA
	Ctenophora	0.06	N	NA	NA
	Cnidaria	0.07	N	NA	NA
Crustacea	Achelata	0.08	N	0.87	P
	Anomura	0.01	N	0.86	P
	Brachyura	0.09	N	0	0
	Copepoda	0.06	N	0.46	P
	Hoplocarida	0.1	N	0.02	NA
	Dendrobranchiata	0.19	N	0	0
	All Crustacea	0	0	0.12	P
Mollusca^b^	Caenogastropoda	0	N	NA	NA
	Cephalopoda	0.26	N	0.01	0
	Heterobranchia	0.16	N	0.7	P
	Neritomorpha	0	N	0.33	P
	Palaeoheterodonta	0	0	0.15	N
	Pteriomorpha	0	0	NA	NA
	Vetigastropoda	0.02	N	0.21	P
Other Inv	Annelida	0.06	N	NA	NA
	Arachnida	0.22	N	0.01	P
	Nemertea	0.33	N	NA	NA
	Myriapoda	0	0	NA	NA
	Nematoda	0.12	N	NA	NA
Insect	Aedes	0.18	N	0.65	P
	Anopheles	0.03	N	0.59	P
	Aschiza	0.03	0	0.09	P
	Bibionomorpha	0.23	N	0.15	P
	Blattodea	0.01	0	0.77	P
	Coleoptera	0	0	0.79	P
	Culex	0.22	N	0.66	P
	Hemiptera	0.14	N	0.71	P
	Hymenoptera	0.27	N	0.68	P
	Isoptera	0.13	N	0.79	P
	Lepidoptera	0.35	N	0.99	P
	Mantodea	0.15	N	0.7	P
	Mecoptera	0.16	P	0.52	P
	Megaloptera	0.15	N	0.24	P
	Odonata	0.11	N	0.58	P
	Phasmatodea	0.14	N	0.44	P
	Plecoptera	0.44	N	0.14	P
	Psocoptera	0	0	0.64	P
	Siphonaptera	0.05	N	0.56	P
	Thysanoptera	0	O	0.5	P
	Trichoptera	0.19	N	0.9	P
	All Insect	0.18	N	0.92	P

The *p* test results for significant nonzero slopes are shown in the table for the groups we could do comparisons for. H represents heavy (majority) strand, L represents light (minority) strand. P, N and 0 stand for positive, negative, and zero slope respectively.

^a^NA signifies no attempt at correlation, as there were only 0 to 2 genes on L strand. HR = correlation coefficient for heavy strand test. LR = correlation coefficient for light strand test. Hm = sign of slope for heavy strand test. Lm = sign of slope for light strand test.

^b^NA as a result of there being too many strand rearrangements to group molluscan taxa for testing. Numbers in parentheses indicate the number of taxa in the group. The lack of correlation (ie small R^2^) for a group) might indicate true lack of correlation or that our assumed OR is inaccurate.

**Fig. 7.**
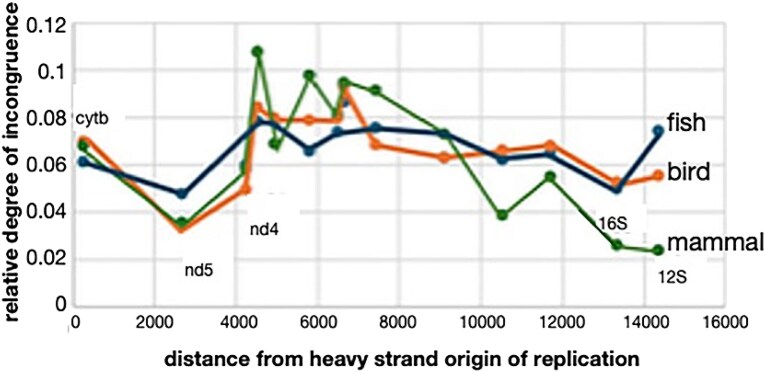
Distance of vertebrate genes from heavy strand OR versus congruence of gene to rest. The ND6 gene is not included in this figure as it is on the light strand.

While the distances of majority strand genes from the OR are not correlated linearly with respect to lack of congruence, the closest few genes to the OR in vertebrates show a distance effect as a result of ND5 and ND4 lacking congruence to other partitions in the mtDNA genome. Figure 7 shows a plot of distance from OR versus degree of incongruence for the 15 vertebrate genes in this study. The figure demonstrates that while CYTb is not the most incongruent gene in the genome as expected by it being nearest to the origin of majority strand replication, the next few genes adjacent to the majority strand OR are highly incongruent in comparison. In addition, we observe that the two rRNA genes that are the most distant genes from the origin of majority strand replication in vertebrates also show low degrees of congruence with the rest of the genes in the mtDNA genome.

## Discussion

Mitochondria are regularly studied for their function (eg ATP production) and as genetic markers for comparing organisms (for phylogenetics, DNA barcoding, and population genetics). Little research has been done to examine how these two major types of studies interact (DeSalle et al. 2017; Rackham and Filipovska 2022; Monzel et al. 2023). Our results strongly suggest that mitochondrial genes often have disparate—incongruent—phylogenetic branching patterns when compared to each other. These results indicate that mitochondrial gene evolution is complex. In practical terms, we suggest that not all mitochondrial genes are created equally for their ubiquitous use in organism identifications and phylogenetics. Our results have bearing on several aspects of mitochondrial DNA utility for biological studies. First, the patterns we observe should help guide further use of the mtDNA genome in phylogenetics. Second, we suggest that the function and organization of animal mitochondrial DNA very much links to how the mitochondrial genes have divergently evolved. In the following, we discuss the four aspects of mtDNA biology that are impacted by the current study.

### The Extent of Incongruence in Animal mtDNA Genomes

While genes in the mtDNA molecule are physically linked as a result of the circular nature of the genome, we found that there is a large degree of incongruent phylogenetic information when such information is compared across genes and taxonomic groups. We observe an unusual lack of phylogenetic congruence among genes in animal mitochondrial genomes (Figs. 4 and 5). The highest pairwise congruence level we observe is for vertebrates (birds and fish) at about 25% to 30% congruence, meaning that over 60% of pairwise gene comparisons result in an observation of incongruence. The lowest we observe is less than 5% of the pairwise comparisons showing congruence. We can rule out dataset size as a major contributing factor to the observed incongruence and attempt to determine if mutational bias or the strand position of the gene is involved in the incongruence. What this result means is that different genes in the mitochondrial genomes of animals carry different phylogenetic signals despite their linked nature. This situation has previously been reported in some plant groups (Gonçalves et al. 2019, 2020; Doyle 2022), insect groups (Campos-Soto et al. 2015; Kim et al. 2020; DeSalle et al. 2022), and other bird groups (Meiklejohn et al. 2014).

### An mtDNA Congruence Gradient

Some researchers have attributed the incongruence of mtDNA genes to mutational gradients; the idea being that as DNA replication gets further from the OR, mutations accumulate more frequently (Faith and Pollock 2003; Sanchez-Contreras et al. 2021), and as mutations accumulate, incongruent phylogenetic information manifests. Another factor might be mutational bias due to nucleotide content in the genome. A final major factor might be differences in the function of the various categories of genes in the mitogenome (synthases, dehydrogenases, oxidases, and rRNAs).

We observe a correlation of degree of incongruence with distance to the OR in invertebrates generally and in insects specifically. This can be observed qualitatively in Fig. 5, where genes that are quite distant from the OR (ND5, ND4, ND1) show the most incongruence. We quantified this general observation by regressing the distance a gene is positioned from the OR for genes on the majority strand and minority strand of vertebrates and on the majority and minority strands of other taxa).

Table 1 shows that there is little correlation with respect to the majority strand versus distance from the OR. In fact, almost all majority strand regressions had negative slopes, but almost entirely with very weak correlation coefficients. However, the general result of Fig. 7 suggests that some of the genes nearest to the majority strand origin show the lowest congruence with others.

In most cases when we could test for a gradient on the minority strand, we obtained reasonably strong correlation coefficients and positive slopes for the regressions. This indicates that the greater the distance from the OR, the more incongruence a gene will show in phylogenetic analysis, with this observation being valid for the minority strand in invertebrates when the ND1, ND4, ND4L, and ND5 genes reside on the minority strand. These results are probably related to strand asymmetry for replication of the mitochondrial genome.

However, it has been pointed out that for the continuously replicated heavy strand for vertebrates the genes nearest the OR for the heavy strand are those that are exposed as single stranded during most of the replication process. This single stranded exposure might also cause a skew in mutational bias and predicts that the closest gene to the OR in vertebrates (cytochrome b) should show the highest degree of mutational bias. We cannot confirm this prediction, but instead observe that the next closest genes on the heavy strand in vertebrate mtDNA are indeed the most incongruent, lending some credence to the idea that close proximity to the vertebrate heavy strand OR results in mutational bias.

In most invertebrates, the rRNAs are adjacent to the OR on the minority strand, which might account for their widespread differences in congruence and nucleotide skew to other genes in the genome. For ND1, ND4, ND4L, and ND5 in invertebrates, these genes are mostly directly opposite of the OR. All other genes are of intermediate distance from the OR. The distance of these genes from the OR might also account for their base composition in invertebrates, a possibility we are currently examining.

### Function and Incongruence

With respect to selection on different functional categories of genes (Rand 2001) as a driver of the large amount of incongruence between mitochondrial genes, we can point out that there seems to be strong congruence among genes within a functional group, but frequent incongruence between functional categories for almost all groups of animals we examined (except for birds and some fish). This result suggests that function (whether a gene codes for an oxidase, a synthase, a dehydrogenase, or a structural RNA) links them together to produce similar phylogenetic patterns. Conversely, we observe a considerable degree of incongruence between genes of different functional categories (Fig. 6). It is difficult to tease apart the role of function of these genes, their nucleotide skew, and their strand location, as each of these aspects appears to link major differences in mitochondrial genes and functional groups. For instance, this difficulty likely arises in part from the fact that the functional categories of the genes in the mitochondria are correlated with their strand location, at least for the minority strand. There are no ATP or COX genes on the minority strand, and the only genes that reside on the minority strand are either rDNAs or ND genes. A full answer to this question will involve some type of multifactor analysis to disentangle the different possible explanations.

### MtDNA in Phylogenetics and Future Studies

It is interesting to note that for vertebrates the one gene found on the light or minority strand (ND6) shows extreme nucleotide mutational bias but is not as incongruent as other genes for vertebrates. In fact, ND5 and ND4 appear to be incongruent with all other partitions in both vertebrates and invertebrates. Incongruence in one group of organisms does not have to be caused by the same mechanism and so we suggest that these patterns of incongruence have different causal backgrounds, and hence should be treated differently in phylogenetic analysis. Conversely, the congruence patterns for ND6 might be caused by its rather short length.

Our results beg the following questions with respect to phylogenetics and mtDNA. First, there are some general questions. If there is rampant incongruence, which gene/genes should be used in phylogenetic analysis when employing mtDNA (Morón-López et al. 2022; He et al. 2023; Main et al. 2024; Menezes et al. 2024)? How do we approach analysis of whole mtDNA genomes in phylogenetic analysis; are partitioning methods the way out? And one specific question about DNA barcoding—in retrospect, has the choice of COXI as a barcode (Hebert et al. 2003; Hebert and Gregory 2005) been prudent or are multiple barcode regions needed? From our analyses, the COX1 gene has a medium degree of incongruence with other genes in the mtDNA genome. This observation suggests that COXI is an appropriate marker for barcoding but that more could be desired.

The answer to these questions may not lie in choosing a gene or genes, but rather in adopting a rather unconventional approach for mtDNA. This approach has been taken up by several research groups using organellar DNA as a source of phylogenetic information and involves the application of multispecies coalescence methods (Gonçalves et al. 2019, 2020; Kim et al. 2020). These methods purport to correct for some of the evolutionary processes that cause incongruence. The current study offers a stepping off point for patterns of phylogenetic results that would be relevant to methodological decision making for mtDNA phylogenetic analysis.

## Materials and Methods

### Sequences and Phylogenetic Matrix Construction

Mitochondrial genome sequences for 89 taxonomically well-defined groups of animals were collated from GenBank using the Organelle webtool or the Nucleotide search engine at NIH/NCBI. We attempted to eliminate any overlap and/or nesting of taxa in constructing the phylogenetic matrices we assembled. Cameron (2025) pointed out that modern annotation processes have made the job of constructing insect mtDNA data matrices more accurate, despite errors in annotation and the trend to submit unannotated mtDNA genomes to GenBank. While we did not reannotate the mitochondrial genomes we used, we scrutinized the genomes included in our study by eliminating entries in the phylogenetic matrices that were clearly misannotated based on visual inspection of alignments. We did not use unannotated genomes at the gene level, as our pipeline for matrix construction required gene annotation for any gene to be incorporated into a matrix for this study.

Table 2 lists all of the taxa, number of taxa in each group, taxonomic rank, common name, and GenBank taxonomic ID. All accession numbers of sequences used in this study are given in Supplementary File S1. We tried to incorporate ordinal-level taxonomic groups (75 groups are either ordinal or sub-, supra-, or infra- order) but occasionally kept phylum-level (Cnidaria, Ctenophora, Porifera, and Placozoa) or subphylum-level (Myriapoda) groups in the analysis to increase the breadth of the analysis. Some groups were categorized at the “clade”–level, which in all six cases are subdivisions of an order. In essence, the only groups in our study that have taxonomic ranks greater than order are the phyla Cnidaria, Placozoa, Porifera, and Ctenophora.

**Table 2:** List of the 89 taxonomic groups used in this study

Abbrev	Scientific name	Common name	Taxonomic ID	Rank	ntaxa
achel	Achelata	Crustacean	6730	Infraorder	38
aclpe	Acipenseridae	Fish	7900	Order	508
aedes	Aedes	Insect	7158	Subclass	352
afrot	Afrotheria	Mammal	311790	Superorder	46
alepo	Alepocephaliformes	Fish	1489486	Order	142
angul	Anguilliformes	Fish	7933	Order	67
annel	Annelida	Worm	6340	No rank	202
anomu	Anomura	Crustacean	6738	Order	118
anoph	Anopheles	Insect	7164	Order	190
anura	Anura	Amphibian	8342	Order	795
arach	Arachnida	Spider	6854	Order	191
asciz	Aschiza	Insect	43737	Clade	46
astro	Asterozoa	Echinoderm	7587	Order	40
atlan	Atlantogenata	Mammal	40674	Order	85
bibni	Bibionomorpha	Insect	43784	Order	71
blatt	Blattodea	Insect	85823	Subphyla	99
bovld	Bovidae	Mammal	9895	Phyh	43
brach	Brachyura	Crustacean	6752	48	
branc	Branchiostoma	Branchiostoma	7737	Order	120
bucer	Bucerotiformes	Bird	57379	Order	69
caeno	Caenogastropoda	Mollusc	69555	Superorder	106
carch	Carcharhiniformes	Fish	30483	Superorder	278
carni	Carnivora	Mammal	33554	Clade	39
cepha	Cephalopoda	Mollusc	6605	Order	760
cetac	Cetacea	Mammal	9721	Order	45
chara	Charadriiformes	Bird	8906	Order	46
chlma	Chimaeriformes	Fish	7864	Order	55
chiro	Chiroptera	Mammal	9397	Phylum	56
chond	Chondrichthyes	Fish	7777	Order	276
chrac	Characiformes	Fish	7991	Order	176
chrom	Chromadorea	Nematode	119089	Superfamily	118
clup	Clupeiformes	Fish	32446	Clade	285
cnlda	Cnidaria	Cnidarian	6073	Phylum	14
coleo	Coleoptera	Insect	7041	Order	30
colum	Columbiformes	Bird	8929	Order	345
copep	Copepoda	Crustacean	6830	Order	636
corac	Coraciiformes	Bird	8936	Infraorder	117
croc	Crocodylia	Crocodile	1294634	Order	35
cteno	Ctenophora	Ctenophora	10197	Order	23
culex	Culex	Insect	7174	Order	55
cypri	Cyprinidae	Fish	7953	Order	360
dendr	Dendrobranchiata	Crustacean	6684	Order	199
eleut	Eleutheria	Cnidarian	13049	Order	219
enop	Enoplea	Nematode	119088	Subclass	83
fatco	Falconiformes	Bird	8948	Order	89
hemip	Hemiptera	Insect	7524	Order	508
heter	Heterobranchia	Mollusc	216305	Subclass	352
hoplo	Hoplocarida	Crustacean	75389	Superorder	46
hymen	Hymenoptera	Insect	7399	Order	142
insec	Eulipotyphla	Mammal	9362	Order	67
lsopt	Termitoidae	Insect	1912919	No rank	202
lago	Lagomorpha	Mammal	9975	Order	118
lamnl	Lamniformes	Fish	30496	Order	190
lepid	Lepidoptera	Insect	7088	Order	795
manto	Mantodea	Insect	7504	Order	191
marsu	Metatheria	Mammal	9263	Clade	46
mecop	Mecoptera	Insect	27420	Order	40
megal	Megaloptera	Insect	50553	Order	85
mylio	Myliobatiformes	Fish	117851	Order	71
myria	Myriapoda	Invertebrate	61985	Subphyla	99
nemer	Nemertea	Ribbon worms	6217	Phylum	43
nerlt	Neritimorpha	Mollusc	119J97	Subcl	48
odon	Odonata	Insect	6961	Order	120
osteo	Osteoglossiformes	Fish	41712	Order	69
paleo	Palaeognathae	Bird	8783	Superorder	106
paleoh	Palaeoheterdonta	Mollusc	47520	Superorder	278
pelme	Pelmatozoa	Echinoderm	133550	Clade	39
perci	Perciformes	Fish	8111	Order	760
phasm	Phasmatodea	Insect	7020	Order	45
pictif	Piciformes	Fish	9219	Order	46
pleco	Plecoptera	Insect	50622	Order	55
porlf	Porifera	Sponge	6040	Phylum	56
prima	Primates	Mammal	9443	Order	276
psltt	Psittaciformes	Bird	9223	Order	176
psoco	Psocodea	Insect	1930602	Superfamily	118
pterl	Pteriomorphia	Mollusc	6545	Clade	285
pzoa	Placozoa	Placozoa	10226	Phylum	14
rhino	Rhinopristiformes	Birds	117887	Order	30
roden	Rodentia	Mammal	9989	Order	345
salmo	Salmoniformes	Fish	8006	Order	636
serpe	Serpentes	Snake	8570	Infraorder	117
siph	Siphonaptera	Insect	7509	Order	35
squal	Squaliformes	Listi	282414	Order	23
strig	Strigiformes	Bird	30458	Order	55
test	Testudines	Turtles	8459	Order	360
thys	Zygentoma	Insect	30264	Order	199
trlch	Trichoptera	Insect	30263	Order	219
vetig	Vetigastropoda	Mollusc	216275	Subclass	83
xenar	Xenarthra	Mammal	9J48	Order	89

Throughout this paper, the groups used are designated by abbreviations seen in the first columns of the table. Taxonomic ID indicates the GenBank taxonomic ID number, and ntax indicates the number of accessions in each higher group used in matrix construction.

Several higher order groupings of the ordinal-level groups we used are possible. For instance fish, birds, and mammals can easily be grouped together as vertebrates at this higher level. Likewise, the 21 insect orders we included can be grouped as insects to establish a higher order grouping for these taxa. We can also subdivide the larger group of insects further into holometabolous and hemimetabolous insects. In some of our analyses and figures, we use the designation “vertebrates” with confidence as this group is monophyletic. We use the designation “invertebrates” with caution as invertebrates are not a monophyletic group. Echinodermata (a deuterostome) are the prime example of “invertebrate” grouping not being monophyletic with the rest of invertebrates. The paraphyly of Cnidaria, Placozoa, Porifera, and Ctenophora with respect to all other animals (the combination of protostomes and deuterostomes) is a second example of non-monophyly when using the term “invertebrate.” We therefore attempt to discern between the deuterostome invertebrates, the protostome invertebrates, and the non-bilateral invertebrates.

Each dataset was partitioned into the 13 protein coding regions and the 2 rRNA genes in the mitogenome. This allowed us to compare the different gene partitions in the mitogenome. While many phylogenetic studies utilize amino acid translations of the proteins, we decided to focus on the nucleotide level because we could then directly compare the patterns for protein coding genes with the two rRNAs encoded in the mitogenomes of animals. Sequences were aligned using MAFFT Version 7 (Katoh and Standley 2013). While this alignment procedure is not codon aware (it can produce stretches of gaps that are not multiples of three—the length of a codon), it was used for four reasons: (i) We wanted to keep our alignment procedures consistent over the 15 genes in this study (2 of which, again, are nonprotein coding and not appropriate for codon-aware alignment). (ii) We did not use a codon-based model for phylogenetic analysis, but rather a site by site model and hence the codon positions do not add information to the models used. (iii) Examination of alignments and tests for the impact of “gappiness” on phylogenetic hypothesis (see below) indicate that the alignments generated for this study were stable with minimum impact of gap insertions via alignment. (iv) Upon visual inspection, we found that most alignments retained triplet organization and hence we reasoned that codon-aware alignment would not add to the analysis. In addition, we directly compared the results of five groups chosen for their range of gappiness (Acipenseridae, Afrotheria, Aschiza, Copepoda, and Columbiformes) for agreement of results using codon-unaware alignments and codon-aware alignments (Supplementary File S2) and observed no significant difference in the patterns we report here.

We make a distinction here between “phylogenetic” matrices (those matrices with the sequence information in them) and “summary” matrices (with the results of the likelihood incongruence tests in them). This step resulted in summary matrices with 15 × 15 genes dimension (with the diagonal being ones, as these are self-comparisons and by definition not incongruent with each other). The matrices for each taxonomic group ranged in size from 25 to over 1,000 taxa with an average taxon set size of 193 (Table 2). Pairwise bp-RELL, p-KH, p-SH, p-wKH, p-wSH, c-ELW, and p-AU tests were then generated for each taxonomic group.

#### Construction of “Antigene” Matrices

All partitions of the mitochondrial genome show a degree of incongruence when tested for the 89 datasets we assembled. We reasoned that if a single gene is removed from a whole genome matrix and the resulting “antigene” phylogenetic matrix tested for incongruence with individual gene matrices shows a lack of incongruence, then we assume that the removed partition contributes significantly to any incongruence of the entire set of genes in the mitogenome. In other words, if a full dataset of 15 partitions is tested for incongruence among all partitions and the test infers incongruence, we do not know which gene or combinations of genes causes the incongruence. To tease this apart, we reasoned that if a partition or combination of partitions were removed and the incongruence tests result in rejection of incongruence amongst the partitions, then the removed partition was the cause of the incongruence. There are other permutations of inclusion and exclusion, which we did not explore such as “antigene by antigene” or “antigene by whole genome” that we did not examine.

The antigene matrices were constructed by removing a single gene partition from the total 15 (an antigene arrangement such that, for instance, anti-COX1 indicates a matrix with COX1 missing and all other genes present). A new summary matrix based on likelihood comparisons was then constructed for each of the 89 taxonomic groups. We next compared the resulting set of antigene matrices for congruence with single gene matrices in the dataset (gene-by-antigene comparisons). This procedure resulted in 15 antigene by 14 gene matrices with congruence measures for each taxonomic group. In other words, each of the 15 antigene phylogenetic matrices is compared to the 14 single gene matrices that are not itself. Comparing an antigene matrix, say for COX1 with the single gene, COX1 phylogenetic matrix is excluded from the summary matrix and so on for all of the 15 genes, leaving 14 comparisons for each of the 15 genes.

### Phylogenetic Approaches—Tree Building Methods

We used maximum likelihood (ML) as implemented in IQTree Version 2 (Nguyen et al. 2015; Minh et al. 2020) to generate all phylogenetic trees (except for our initial analyses of ILDs where we used the parsimony criterion in PAUP). We explored the model space by allowing IQTree to determine the best model for a subset of the matrices we used. The majority of these tests resulted in the choice of the TIM2 model with G and I as optimal parameters. To produce internally consistent results, we applied the TIM2 + G + I model in all phylogenetic analyses we present here. The TIM2 model attempts to correct for transition bias, which mostly occurs in the third position of codons. Tree searches were accomplished with the default tree search setting in IQTree, which start with a random tree and a hill-climbing algorithm, which iteratively optimizes the random tree by branch-swapping.

### Phylogenetic Methods—Tests of Incongruence

#### Rationale and Flow of Analyses for Incongruence

We first examined the quality of inferences we can make using the incongruence tests, comparing parsimony-generated ILD statistics with the likelihood-generated incongruence inferences. Since we find a strong positive correlation of ILD test results with the likelihood tests (not shown), we focus on likelihood methods in the rest of the paper.

We used PAUP (Swofford 1993) and IQTree Version 2 (Nguyen et al. 2015; Minh et al. 2020) to generate all phylogenetic trees and to test for incongruence. In PAUP, pairwise comparisons of incongruence between two partitions were calculated using the ILD test (a parsimony-based comparison of tree lengths). We also used IQTree Version 2 to conduct likelihood-based incongruence tests (bp-RELL, p-KH, p-SH, p-WKH, p-WSH, c-ELW, and p-AU; p stands for p value and w stands for weighted estimate), which give probabilities that two trees (inferred from single genes or any combination of genes) are incongruent. An incongruence test for two genes involves the use of the likelihood of the topologies for the trees inferred individually with the alignment for one of the genes. For instance, if we compare COXI with COXII for a specific taxonomic dataset, the likelihoods of the tree topologies for COXI and COXII are compared to the COXI alignment. One can also compare the likelihoods of tree topologies for COXI and COXII to the COXII alignment. We used the same substitution model (TIM2 + G + I) in all comparisons and the same search parameters for all incongruence comparisons. If both partitions significantly reject the other's tree, then there is strong incongruence between the two partitions. Conversely, if neither can reject incongruence, then the two genes or partitions being tested are either truly congruent as a result of congruent signal in the different partitions or the result is ambiguous (the signal in one or both of the genes is too weak to result in a significant inference). These latter cases are more realistic and can occur when one or both of the compared gene regions or partitions are small as in the ND4L and ATP8 gene regions, which we discuss in the Results section of this paper.

#### Data Set Size and Incongruence

Knowing that the number of taxa in an analysis impacts the incongruence of any tree generated from a matrix to others (Sanderson and Donoghue 1989; O’Grady et al. 2001), we examined the impact of taxon set size on incongruence measures. To do this, we took our four largest matrices with respect to number of taxa from the major groups in this study (insects, Crustacea, mammals, and mollusks) and randomly partitioned them into matrices that were approximately 50%, 25%, and 10% their original size by culling away the appropriate percentage of taxa. The matrices represented are Blattoidea (insect; ntax = 1,009, 500, 250, 100), Rodentia (mammals; ntax = 324, 150, 75, 30), Caenogastropoda (Mollusca; ntax = 555, 250, 125, 55), and Brachyura (Crustacea; ntax = 409, 200, 85, 40). We then compared the congruence inferences from these matrices with randomly chosen taxa with the full matrix.

#### Regression of Distance from the OR on Unscaled Incongruence Measures

The general location of the OR is known in most vertebrate taxa and a considerable number of invertebrate protostome taxa. Conversely, the OR is not known in a significant number of invertebrate genomes. For those genomes where the OR is known, we computed an unscaled incongruence measure for each gene for the various groups in the study. To do this, we simply counted the number of gene-by-matrix comparisons for each gene by each dataset that are incongruent with the rest. This metric is simple and easy to compute and gives values that quantify the number of comparisons a specific gene partition is incongruent with all others. We compiled the distances of the 15 genes used in this study from the OR and used these distances to test for correlation with unscaled incongruence measures. All regressions were performed in XLSTAT, a plugin program for Excel.

## Supplementary Material

evaf209_Supplementary_Data

## Data Availability

All datasets and analyses are available at Data Dryad (DOI: 10.5061/dryad.4f4qrfjqf).

## Supplementary Material

Table S1. All the taxa and accession numbers used in this study.

Supplementary File S1. All the incongruence measures used to generate heatmaps in Figs. 3 to 6.

Supplementary File S2. A comparison of alignment aware analyses with alignment agnostic analyses of five randomly chosen matrices.

Supplementary File S3. All the 89 phylogenetic matrices of aligned and partitioned sequences used in this study. It is available on Data Dryad (DOI: 10.5061/dryad.m0cfxpp9m).
